# Role of the VEGF ligand to receptor ratio in the progression of mismatch repair-proficient colorectal cancer

**DOI:** 10.1186/1471-2407-10-93

**Published:** 2010-03-11

**Authors:** Manuela Eppenberger, Inti Zlobec, Daniel Baumhoer, Luigi Terracciano, Alessandro Lugli

**Affiliations:** 1 Institute of Pathology, University Hospital of Basel, Basel, Switzerland

## Abstract

**Background:**

The VEGF family of ligands and receptors are intimately involved in tumor angiogenesis, lymphangiogenesis and metastasis. The evaluation of VEGF ligand/receptor ratios may provide a more profound understanding of the involvement of these proteins in colorectal tumour progression. The aim of this study was to elucidate the role of the VEGF ligand/receptor ratios on tumour progression and metastasis in patients with mismatch repair-proficient colorectal cancer.

**Methods:**

Immunohistochemistry for VEGF-A, VEGF-B, VEGF-C, VEGF-D, VEGFR1, VEGFR2 and VEGF3 was carried out on 387 mismatch repair-proficient colorectal cancers using a tissue microarray. Evaluation of immunoreactivity was performed semi-quantitatively and the ligand/receptor expression ratio was obtained.

**Results:**

An increased VEGF-A/VEGFR1 ratio, VEGF-A and VEGFR1 was linked to the presence of peritumoral lymphocytic inflammation at the invasive front (p = 0.032; p = 0.005; p = 0.032, respectively). VEGFR1 expression was related to poorer outcome in multivariable analysis with pT stage, pN stage, vascular invasion, and post-operative therapy. A higher ratio of VEGF-A/VEGFR2 was linked to advanced TNM stage (p = 0.005) while VEGF-A and VEGFR2 were elevated in tumours with an infiltrating tumour growth pattern (p = 0.006; p = 0.014; p = 0.006). No effect of VEGF-A/VEGFR2, VEGF-A or VEGFR2 on survival time was noted.

**Conclusions:**

Our findings highlight an involvement of VEGF-A, VEGR1 and VEGFR2 in events occurring at the invasive tumour front and a potential prognostic role of VEGFR1 expression in mismatch repair-proficient colorectal cancers. The VEGF-A ligand to VEGFR1 or VEGFR2 ratio may represent an alternative evaluation system for identifying patients with poorer clinical outcome.

## Background

Angiogenesis, the process of developing new blood vessels from pre-existing vascular networks, is now a well-described mechanism leading to the initiation and maintenance of tumours, and the promotion of metastasis at secondary sites [1]. Hypoxia is a major activator of angiogenesis in tumours [2]; the hypoxic state of cells promotes the up-regulation of a variety of cytokines and tumour suppressors, such as p53 and also of hypoxia-inducible factor 1-alpha, primarily known for its ability to activate Vascular Endothelial Growth Factor (VEGF) expression [3].

The VEGF family of ligands and receptors includes VEGF-A, VEGF-B, VEGF-C, VEGF-D, platelet derived growth factor (PlGF) and VEGFR1, VEGFR2, VEGFR3 and neuropilin NP1 and NP2 [4]. The best characterized of the VEGF family members is VEGF-A, whose binding to VEGFR2 (FLK1) is the predominant mechanism through which tumour cells promote angiogenesis. VEGF-A/VEGFR2 binding activates RAS/RAF-1/MEK/ERK phosphorylation as well as signalling through PI3K/pAKT. In response to signalling activity, up-regulation of downstream effectors such as mdm2, p53, p27, endothelial nitric oxide, and Bcl-2 can occur as well as inhibition of pro-apoptotic proteins caspase-9 and APAF-1. The consequences of this binding are increased vascular permeability, enhanced endothelial cell proliferation as well as increased survival, migration and invasion of tumour cells. Although significantly less is known about VEGFR1 (FLT1), it appears to function as a negative regulator of angiogenesis [5]. VEGF-A is expressed on vascular cells and binds to VEGFR1 with an affinity that is much higher than that for VEGFR2. However, VEGFA seems to induce much weaker tyrosine kinase activity in VEGFR1 possibly because of an inhibitory sequence in the juxtamembrane domain that represses VEGFR1 activity [6]. In keeping with this observation, a model for VEGFR1 has been developed whereby it could act as a decoy receptor to modulate angiogenesis through its ability to sequester VEGFA thereby reducing signaling through VEGFR2. VEGF-B has also been found to bind to VEGFR1, although the role of this interaction remains to be completely elucidated. VEGFR3 is the specific receptor for VEGF-C and -D and is predominantly found on lymphatic, but also to a lesser extent, on vascular endothelial cells and also on tumour cells [7]. Interestingly, VEGF-C along with VEGF-A and a variety of pro-angiogenic cytokines have been shown to be released from tumour associated macrophages, whose infiltration is thought to be, at least in part, responsible for the angiogenic switch in tumours whereby the balance of pro- and anti-angiogeneic factors favour a pro-angiogenic phenotype [8-10].

In 1971, the pioneering work by Folkman and colleagues led to the hypothesis that anti-angiogenic compounds could be successfully applied as anti-cancer therapies [11,12]. In fact, blocking of VEGF has been shown to lead to normalization of the vasculature, thus increasing the efficacy of both radiotherapy (by increasing the partial oxygen pressure of cells) and also the delivery of chemotherapeutic agents to target cells (by decreasing vascular permeability) [13]. Currently, the humanized monoclonal antibody Bevacizumab approved for the treatment of patients with metastatic colorectal cancer has been successful in improving overall survival times in several randomized controlled studies while other approaches such as the use of tyrosine kinase inhibitors continue to be investigated [14,15]. VEGFR1 immunoreactivity in tumour cells has been correlated with poor prognosis, metastasis and recurrence in a variety of tumour types including breast and lung cancers [16-18]. Inhibitors of VEGFR1 activity, such as VEGFR1 antibodies or soluble VEGFR1 traps have been developed for preclinical and clinical evaluation and have been shown to suppress tumour growth by inhibiting expression of VEGF on both tumour and stromal cells [5].

Although several studies have evaluated one or more of these VEGF ligands or their receptors by immunohistochemistry and their potential prognostic value, still lacking is a comprehensive analysis performed on a large number of tumours from patients with full clinico-pathological data taking into consideration the different expression ratios between the VEGF ligands and their receptors. Such an evaluation may provide a more profound understanding of the involvement of these angiogenic proteins in colorectal tumour progression, particularly considering the known differences in binding affinities of VEGF ligands to their receptors. The aim of this study was therefore to elucidate the prognostic role of the VEGF ligand to receptor ratios and their effects in tumour progression and metastasis on 387 patients with mismatch repair-proficient colorectal cancers.

## Methods

### Patients

489 non-consecutive, unselected colorectal cancer patients treated at the Stadtspital Triemli, Zürich, Switzerland were initially entered into this study. Histomorphologic features were systematically re-reviewed from the corresponding hematoxylin and eosin (H&E) stained slides in all cases and included TNM stage (according to the 6^th ^edition of the American Joint Committee on Cancer staging manual), tumor grade, the presence of vascular invasion, the tumor border configuration, the presence of peritumoral lymphocytic infiltration, the histologic tumor subtype and mismatch repair status. Patients with mismatch repair-deficient tumours (n = 102) were excluded from the study. Clinical data were retrieved from the patient records and included age, gender, tumor location, follow-up and disease-specific survival time, information on local recurrence and distant liver metastasis. Of the 387 patients with mismatch repair-proficient colorectal cancers, 88 received post-operative therapy. All patients were pre-operatively untreated and all resections were complete. Patient characteristics are summarized in Table 1.

**Table 1:** Characteristics of patients with mismatch repair-proficient colorectal cancer

Clinico-pathological feature		Frequency n (%)
Gender (n = 381)	Female	175 (45.9)
	Male	206 (54.1)
		
Tumour location (n = 369)	Left-sided	242 (65.6)
	Right-sided	127 (34.4)
		
pT stage (n = 373)	pT1-2	86 (23.1)
	pT3-4	287 (76.9)
		
pN stage (n = 357)	pN0	178 (49.9)
	pN1-2	179 (50.1)
		
Tumour grade (n = 374)	G1-2	289 (77.3)
	G3	85 (22.7)
		
Vascular invasion (n = 374)	Absent	267 (71.4)
	Present	107 (28.6)
		
Tumour border configuration (n = 374)	Pushing	195 (52.1)
	Infiltrating	179 (47.9)
		
Peritumoural lymphocytic infiltration (n = 374)	Absent	308 (82.4)
	Present	66 (17.7)
		
Local recurrence (n = 377)	Absent	208 (55.2)
	Present	169 (44.8)
		
Liver metastasis (n = 387)	Absent	313 (80.9)
	Present	74 (19.1)
		
Post-operative therapy (n = 377)	No	289 (76.7)
	Yes	88 (23.3)
		
Survival time (n = 377)	5-year (%) (95%CI)	58.9 (52-65)

*G1-G2: well and moderately differentiated tumours; G3: poorly differentiated tumors

### Specimen characteristics

Formalin-fixed paraffin-embedded tissue blocks were retrospectively retrieved from the archives of the Institute of Pathology, Stadtspital Triemli, Zürich, Switzerland and a tissue microarray including all 387 patients was constructed from each colorectal cancer resection specimen. From each patient, one representative tumor block was punched from the tumor centre using a tissue cylinder 0.6 mm in diameter. Tissue was brought into one recipient paraffin block (3 × 2.5 cm) using a homemade semi-automated tissue arrayer as described elsewhere [19] 71 tissues from normal colorectal mucosa were included as a control.

### Assay Methods

#### Immunohistochemistry

Immunohistochemistry for VEGF ligands and receptors was carried out. Briefly, the tissue microarray was dewaxed and rehydrated in dH_2_O. Following pressure cooker mediated antigen retrieval in 0.001 M ethylenediaminetetraacetic acid (EDTA) pH 8.0, endogenous peroxidase activity was blocked using 0.5% H_2_O_2 _and the sections were incubated with 10% normal goat serum (Dako Cytomation, Carpinteria, CA) for 20 min. The colorectal cancers were then incubated with primary antibody for for VEGF-A (Santa Cruz; USA; 1:300), VEGF-B (Santa Cruz; USA; 1:100), VEGF-C (Santa Cruz; USA; 1:100), VEGF-D (Santa Cruz; USA; 1:100), VEGFR1 (LabVision; USA; 1:100), VEGFR2 (Santa Cruz; USA; 1:1000) and VEGFR3 (Chemicon; USA; 1:200). Subsequently, sections were incubated with HRP-conjugated secondary antibody (DakoCytomation) for 30 min at room temperature. For visualization of the antigen, the sections were immersed in 3-amino-9-ethylcarbazole+substrate-chromogen (DakoCytomation) for 30 min, and counterstained with Gill's Haematoxylin. Negative controls underwent the identical procedure with primary antibody omitted. Positive controls consisted of tumours known to contain the protein of interest. Additionally, internal positive controls for VEGF ligands, such as inflammatory cells, needed also to be present for immunohistochemistry to be considered valid. The use of tissue for this study was approved by the local Ethics Committee of the University Hospital of Basel.

#### Evaluation

**Figure 1:**
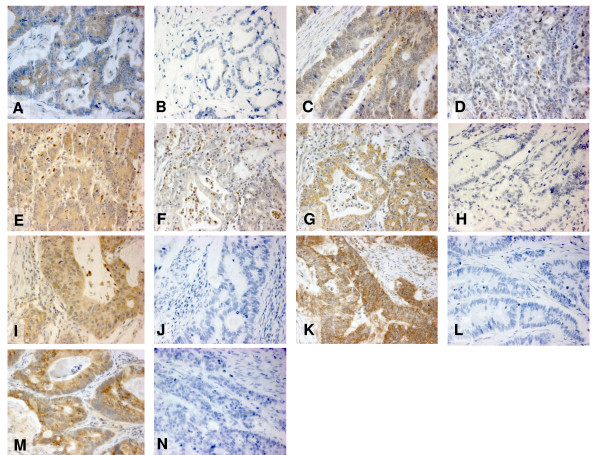
**Figure 1A-N**. Different colorectal cancer samples with negative and positive staining for VEGF ligands (cytoplasmic) and receptors (membraneous and/or cytoplasmic): VEGFA+ (A), VEGFA- (B), VEGFB+ (C), VEGFB- (D), VEGFC+ (E), VEGFC- (F), VEGFD+ (G), VEGFD- (H), VEGFR1+ (I), VEGFR1- (J), VEGFR2+ (K), VEGFR2- (L), VEGFR3+ (M) and VEGFR3- (N).

The evaluation of immunohistochemistry was performed by an expert gastro-intestinal pathologist blinded to patient outcome. Using a semi-quantitative scoring method which describes the percentage of positive tumour cells assigned to each case on 5% intervals (from 0% to 100%). This scoring system has previously been found to lead to significant inter-observer agreement between independent pathologists, particularly using tissue microarrays. Staining intensity for most VEGF ligands and receptors was homogeneous and therefore not assessed. For VEGF ligands, cytoplasmic staining was assessed whereas receptor expression was scored in both cytoplasm and membrane. Representative immunostains for VEGF ligands and receptors are illustrated in Figure 1.

#### VEGF ligand/VEGF receptor ratio

VEGF ligand to receptor ratios were determined by utilizing the percentage of immunoreactivity in both cases and dividing expression of the ligand by that of the receptor. The following ratios were explored: VEGF-A/VEGFR1, VEGF-A/VEGFR2, VEGF-B/VEGFR1, VEGFR-C/VEGFR2, VEGFR-C/VEGFR3, VEGFR-D/VEGFR2 and VEGF-D/VEGFR3. In the event that the expression of the receptor was 0% which occurred in 40 cases for VEGFR1, 4 cases for VEGFR2 and 40 cases for VEGFR3, cases were removed from the study. Ratios >1.0 indicate a higher expression of ligand compared to receptor while ratios <1.0 describe tumours with greater expression of receptor compared to ligand.

### Study Design

**Figure 2:**
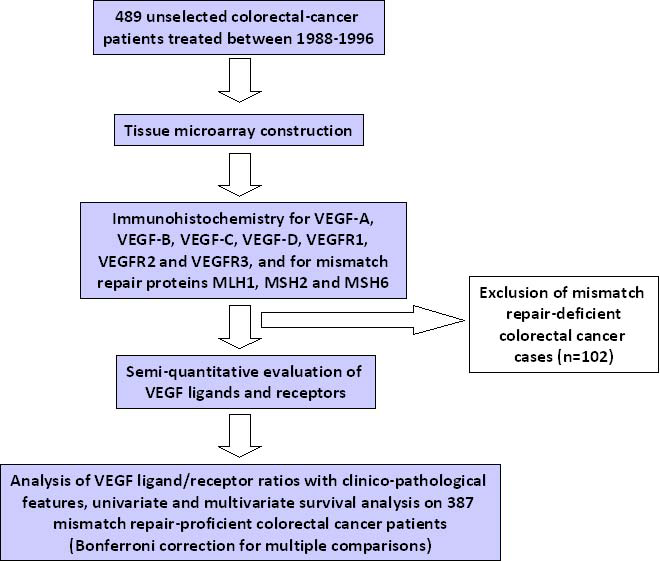
**Study Design**. 489 unselected patients were initially entered into this study. After tissue microarray construction, immunohistochemistry for VEGF ligands and receptors, 102 mismatch repair-deficient cases were excluded. Analysis of VEGF ligand/receptor ratio with clinico-pathological features and survival was performed on the remaining 387 mismatch repair-proficient cases.

The study design is outlined in Figure 2. Resection specimens from 489 patients treated between 1988 and 1996 were collected, retrospectively. A tissue microarray containing all cases was constructed. Immunohistochemistry for VEGF ligands and receptors was carried out and staining evaluated. Immunohistochemistry for mismatch repair markers, MLH1, MSH2 and MSH6 identified 102 cases of mismatch repair-deficient colorectal cancers which were excluded from the analysis. The median follow-up time of patients with mismatch repair-proficient tumours was 30 months (range 0 to 144 months), mean time between local recurrence and surgery was 20.1 months. Outcome measure of interest was cancer-specific survival time.

### Statistical analysis

VEGF ligand/receptor ratios and their association with categorical clinico-pathological features was performed using the non-parametric Wilcoxon Rank Sum Test or Kruskal Wallis test, where appropriate. Missing clinico-pathological information was assumed to be missing at random. Survival analysis was carried out using the Kaplan-Meier and log-rank test. The assumption of proportional hazards was verified by analyzing the correlation of Schoenfeld residuals and the ranks of individual failure times. Subsequently, multivariable Cox proportional hazards regression analysis was carried out to determine the independent prognostic effect of each VEGF ligand, receptor or ratio with significant effects in univariate analysis, after adjusting for the effects of pT, pN and vascular invasion. Hazard ratios (HR) and 95% confidence intervals (CI) were used to determine the prognostic effect of each variable. Due to multiple testing, a Bonferroni correction for multiple comparisons indicated that only p-values < 0.0012 should be considered statistically significant. All analyses were carried out using SAS (V9, The SAS Institute).

## Results

### Comparison of normal mucosa and colorectal cancer

**Figure 3:**
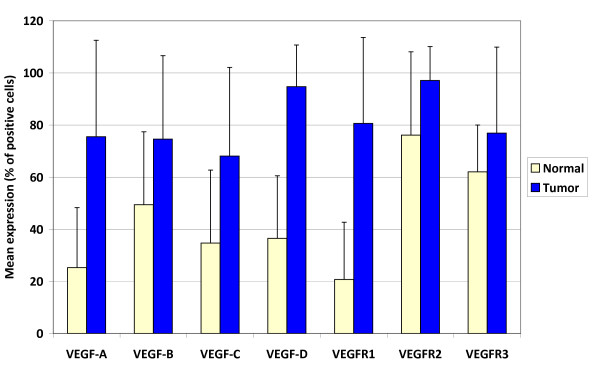
**Differences in mean VEGF ligand and receptor expression in normal colorectal mucosa in comparison to colorectal cancers**. Standard error bars are shown.

Expression of all VEGF ligands and receptors was significantly greater in colorectal cancers compared to normal colorectal mucosa (p < 0.001; Figure 3).

### VEGF ligand/receptor ratios (Tables 2 and 3)

**Table 2:** VEGF-A/VEGFR1, VEGF-A/VEGFR2, VEGF-B/VEGFR1, and VEGF-C/VEGFR2 ratios and association with clinico-pathological features in mismatch repair-proficient colorectal cancer.

		VEGF-A/VEGFR1		VEGF-A/VEGFR2		VEGF-B/VEGFR1		VEGF-C/VEGFR2	
			P-value		P-value		P-value		P-value
TNM stage	I+II	13.7 ± 32.6; 1.0	0.198	1.63 ± 8.6; 1.0	0.005	11.2 ± 27.2; 1.0	0.867	2.6 ± 9.3; 0.8	0.003
	III	13.0 ± 31.7; 1.0		0.66 ± 0.4; 0.95		8.1 ± 23.2; 1.0		0.58 ± 0.38; 0.68	
	IV	5.2 ± 19.7; 1.0		3.85 ± 16.3; 1.0		5.9 ± 20.0; 1.0		1.95 ± 8.8; 0.84	
									
Tumour grade	G1-2	10.7 ± 29.2; 1.0	0.704	2.05 ± 10.6; 1.0	0.179	8.0 ± 22.9; 0.98	0.956	1.51 ± 8.2; 0.79	0.06
	G3	13.5 ± 32.2; 1.0		0.73 ± 0.4; 0.95		12.9 ± 29.7; 1.0		0.58 ± 0.4; 0.65	
									
Vascular invasion	Absent	13.2 ± 32.2; 1.0	0.276	2.2 ± 11.3; 1.0	0.38	10.7 ± 26.9; 1.0	0.078	1.6 ± 8.9; 0.75	0.412
	Present	6.48 ± 22.0; 1.0		0.78 ± 0.36; 0.98		4.28 ± 15.2; 0.95		0.65 ± 0.38; 0.75	
									
Tumour border configuration	PM	12.4 ± 31.2; 1.0	0.211	1.35 ± 6.3; 1.0	0.006	9.8 ± 25.2; 1.0	0.244	1.2 ± 5.7; 0.79	0.326
	IM	9.83 ± 27.6; 1.0		2.28 ± 12.2; 0.95		7.76 ± 23.2; 0.95		1.48 ± 9.04; 0.75	
									
PTL infiltration	Absent	9.73 ± 27.6; 1.0	0.032	1.56 ± 8.4; 1.0	0.074	8.09 ± 23.5; 1.0	0.356	1.01 ± 4.5; 0.78	0.235
	Present	17.5 ± 36.6; 1.0		2.83 ± 13.6; 1.0		12.3 ± 27.5; 0.87		2.79 ± 14.7; 0.75	

Mean ± SD, median values. Kruskal Wallis or Wilcoxon Rank Sum Test. PM = pushing/expanding margin; IM = infiltrating margin; PTL = peritumoral lymphocytic.

**Table 3:** VEGF-C/VEGFR3, VEGF-D/VEGFR2, and VEGF-D/VEGFR3 ratios and association with clinico-pathological features in mismatch repair-proficient colorectal cancer.

		VEGF-C/VEGFR3		VEGF-D/VEGFR2		VEGF-D/VEGFR3	
			P-value		P-value		P-value
TNM stage	I+II	6.0 ± 21.0; 0.95	0.265	1.8 ± 9.1; 1.0	0.55	6.6 ± 22.4; 1.03	0.981
	III	3.99 ± 13.4; 0.79		0.94 ± 0.1; 1.0		13.2 ± 32.0; 1.05	
	IV	5.93 ± 19.7; 1.0		4.67 ± 18.8; 1.0		7.14 ± 23.3; 1.05	
							
Tumour grade	G1-2	5.28 ± 18.3; 0.95	0.116	2.46 ± 11.9; 1.0	0.274	8.34 ± 25.4; 1.05	0.317
	G3	4.94 ± 18.1; 0.75		0.96 ± 0.17; 1.0		11.9 ± 30.5; 1.0	
							
Vascular invasion	Absent	4.56 ± 16.8; 0.95	0.928	2.2 ± 10.5; 1.0	0.53	8.75 ± 26.2; 1.0	0.158
	Present	6.74 ± 21.4; 0.95		2.34 ± 11.6; 1.0		9.7 ± 27.1; 1.05	
							
Tumour border configuration	PM	3.9 ± 15.7; 0.95	0.349	1.82 ± 9.04; 1.0	0.172	7.62 ± 24.5; 1.0	0.437
	IM	6.67 ± 20.7; 0.95		2.53 ± 12.3; 1.0		10.5 ± 28.3; 1.05	
							
PTL infiltration	Absent	6.15 ± 20.1; 0.95	0.733	1.96 ± 9.9; 1.0	0.538	10.6 ± 28.9; 1.0	0.575
	Present	1.09 ± 1.5; 0.85		3.18 ± 14.4; 1.0		1.79 ± 3.0; 1.05	

Mean ± SD, median values. Kruskal-Wallis or Wilcoxon Rank Sum Test. PM = pushing/expanding margin; IM = infiltrating margin; PTL = peritumoral lymphocytic.

An increased VEGF-A/VEGFR1 ratio was observed in patients with tumours presenting conspicuous peritumoural lymphocytic inflammation at the invasive front (p = 0.032). When analyzing VEGF-A and VEGFR1 separately, an increase of both VEGF-A (p = 0.005), and VEGFR1 (p = 0.032) was found in tumours with this histological feature in comparison to those with absence of inflammation. In univariate analysis pT, pN and vascular invasion all demonstrated a significant effect on outcome (p < 0.001, each). These features were included into multivariable survival analysis to determine the effect of the VEGF-A, VEGFR1 and VEGF-A/VEGFR1 ratio on prognosis after adjusting for these 3 features. Increased expression of VEGFR1 was linked to poorer outcome, while the remaining established prognostic factors also maintained their independent effect on outcome (Table 4). VEGFR1 expression also was related to poorer prognosis after adjusting additionally for post-operative adjuvant therapy.

**Table 4:** Multiple Cox regression analysis of VEGFR1 adjusting for T stage, N stage, vascular invasion as well as post-operative adjuvant therapy

Feature		HR (95%CI)	P-value	HR (95%CI)	P-value
VEGFR1	Baseline	1.0	0.024	1.0	0.032
	1-unit increase	1.009 (1.001-1.017)		1.009 (1.001-1.017)	
					
pT stage	pT1-2	1.0	<0.001	1.0	<0.001
	pT3-4	5.67 (2.03-15.8)		5.79 (2.07-16.19)	
					
pN stage	pN0	1.0	<0.001	1.0	<0.001
	pN1-2	4.81 (2.53-9.16)		5.1 (2.61-9.97)	
					
Vascular invasion	Absent	1.0	<0.001	1.0	<0.001
	Present	2.82 (2.82-4.75)		2.93 (1.71-5.02)	
					
Post-operative therapy	None			1.0	0.562
	Treated			0.85 (0.49-1.48)	

The ratio of VEGF-A/VEGFR2 was linked to TNM stage (p = 0.005). The average VEGF-A/VEGFR2 ratio was 3.85 in metastatic stage IV cases compared to 0.66 in stage III and 1.63 in stages I and II. Increased expression of this ratio was again observed in patients with tumours showing an infiltrating tumour growth pattern (p = 0.006). When evaluating VEGF-A or VEGFR2 expression separately and their relationship to the tumour border configuration, a strong loss of VEGF-A (p = 0.014) and VEGFR2 expression in tumours with infiltrating margin (p = 0.006) was observed. However, no effect of VEGF-A/VEGFR2 or VEGFR2 on survival time was noted.

Decreased expression of VEGF-C/VEGFR2 was observed in more advanced TNM stage (p = 0.003). However, the remaining VEGF-ligand/receptor ratios were not related to either clinico-pathological features nor with survival in either metastatic and non-metastatic patients.

## Discussion

This work appears to be the first to evaluate in a single study the immunohistochemical importance of four VEGF ligands with their corresponding receptors in an expression ratio in colorectal cancer. The findings here support a role, not only for VEGF-A, VEGFR1 and VEGFR2 in tumour progression but most importantly of a potential prognostic role of VEGFR1 expression in mismatch repair-proficient colorectal cancer.

The ratio of VEGF-A to VEGFR1 and VEGFR2 as well as the ratio of VEGF-C/VEGFR2 demonstrated the most interesting effects of these angiogenic proteins on progression and survival. These results are similar to those reported by Hanrahan et al. who investigated VEGF ligands and their receptors at the mRNA level in normal, adenoma and colorectal carcinoma [20]. In their study, they suggest that VEGF-A and VEGF-B may be responsible for the initiation of tumour whereas VEGF-A and VEGF-C are further expressed in order to maintain disease progression. They observed a significant correlation between VEGF-A and tumour size but not with tumour stage, lymphovascular invasion or metastasis. In addition, they document a significant link between VEGFR1 expression and tumour grade and Dukes' stage and of both VEGFR1 and VEGFR2 mRNA expression and lymph node positivity. Our findings of an increased VEGF-A expression from normal tissue to tumour, but a lack of association between expression with advanced pT stage, metastasis and survival time further support a role of VEGF-A in initiation and tumour maintenance in colorectal cancer. Furthermore, the combined analysis of VEGF-A with VEGFR1 and their correlation with features of tumour progression and adverse prognosis seem to implicate in particular VEGFR1 and VEGFR2 in the progression of colorectal cancer.

Inflammatory mediators have previously been shown to have a significant effect on the process of angiogenesis through the up-regulation of certain cytokines as well as of VEGF [10,21]. Not only does VEGF increase vascularity at sites of inflammation but its production by tumour cells results in the expression of inter-cellular adhesion molecule-1 and vascular cell adhesion molecule-1, thereby facilitating the adhesion of leukocytes to endothelial cells [22]. Our results highlight a relationship between the over-expression of VEGF-A as well as VEGFR1 and the peritumoural lymphocytic inflammatory response at the invasive tumour front. The inflammatory response at the tumour border has previously been linked to the tumour border configuration, which we recently underlined as an essential prognostic factor in colorectal cancer [23]. The presence of a conspicuous band of lymphocytes, as described by Jass and colleagues is frequently associated with the presence of a pushing tumour margin, and has been related to an increased number of CD8+ tumour infiltrating lymphocytes and to an improved survival time [24,25]. In this study, we find that a greater VEGFR2 expression compared to VEGF-A is possibly linked to the presence of an infiltrating margin. Since an infiltrating tumour border configuration is a histomorphologic feature closely correlated to epithelial mesenchymal transition (EMT), whereby tumour cell de-differentiation and loss of cell-cell adhesion occurs at the invasive tumour front, our results may implicate VEGFR2 in this process [26].

The vast majority of the literature suggest a greater invasion and metastatic phenotype in tumours expressing these proteins [27-33]. In particular, several groups have suggested a VEGFR1-dependent involvement in EMT. Bates and colleagues used a spheroid culture system recapitulating the structure of the colonic epithelium during EMT. Their results find a significant expression of VEGFR1, but not VEGFR2 in these cells [34]. In pancreatic cancer, Yang and coworkers also describe VEGFR1 mediated EMT [35] while in head and neck squamous cell carcinoma (HNSCC), VEGFR2 expression has been linked to vasculogenesis and budding of tumour cells into new vessels [36]. Our results additionally underline not only the expression of VEGF-A as a possible step in tumour progression of colorectal cancer, but more importantly that VEGFR1 and VEGFR2 as well as their ratios with VEGF-A to play a role in the events occurring at the invasive tumour front.

Although VEGF-C and VEGF-D are known primarily as lymphangiogenic proteins, less is known about their prognostic effect in patients with colorectal cancer. Hu and colleagues found that protein expression of VEGF-C and VEGF-D was significantly increased from normal to tumour tissues, a result which we confirm in our study. Furthermore, an increased expression of both these proteins was linked to lymph node metastasis and worse survival time [37]. Kawakami et al. report that VEGF-B and VEGF-C mRNA are significantly higher in tumours with lymph node metastases and in tumours with lymphatic invasion [38] while Onogawa and colleagues report an increased VEGF-C and VEGF-D expression at the invasive tumour front [39]. Others have found a significant association of these proteins with venous and lymphatic invasion as well as with liver metastasis. A recent report by Moehler et al. found that VEGF-D expression correlated with lymph node metastasis and interestingly, that VEGF-D expression was significantly decreased following treatment with anti-EGFR mAb both in vitro and in mouse xenograft models [40]. In our study, a lower expression ratio of VEGF-C/VEGFR2 was linked to more advanced TNM stage.

Our study has several limitations. First it is a retrospective analysis of VEGF ligand and receptor expression and therefore should be investigated in a prospective setting. Secondly, having used the tissue microarray technique, it is possible that tumour heterogeneity was not completely taken into account. As an immunohistochemistry study, inter-laboratory variation may play a role in determining the reproducibility of these findings. Also, having considered adjustment for multiple testing in this study, the associations of VEGF ligands and their receptors fall short of significance although several strong trends were observed. Therefore, our results necessitate confirmation by other, larger study groups. Finally, we were unable to randomize our patient cohort into test and validation subgroups due to the lack of statistical power that this would elicit. Nevertheless, our study may still be a basis for prospective approaches and worth to be validated in future studies.

## Conclusions

The results of this study suggest that the VEGF ligand to receptor ratio may be an informative method for evaluating the effects of these angiogenic proteins on tumour progression. Our findings further underline a potential involvement of VEGF-A, VEGR1 and VEGFR2 in events occurring at the invasive tumour front and highlight a possible prognostic role of VEGFR1 expression in mismatch repair-proficient colorectal cancers.

## Competing interests

The authors declare that they have no competing interests.

## Authors' contributions

ME participated in acquisition of data, data analysis, and manuscript writing. IZ carried out the conception and design, the interpretation of data, and manuscript writing while DB participated in data acquisition and analysis. LT was active in conception and design and AL was responsible for conception and design, data acquisition and analysis, and interpretation of findings. All authors have read and approved the final manuscript.

## Pre-publication history

The pre-publication history for this paper can be accessed here:

http://www.biomedcentral.com/1471-2407/10/93/prepub
